# A multi-layer annotated corpus for information extraction in Russian clinical NLP

**DOI:** 10.3389/frai.2026.1766899

**Published:** 2026-03-31

**Authors:** Anar Sultangaziyeva, Madina Sambetbayeva, Nurzhan Mukazhanov, Bayangali Abdygalym, Sandugash Serikbayeva

**Affiliations:** 1 Department of Science and Cooperation, Q University, Almaty, Kazakhstan; 2 School of Information Technology and Engineering, Astana International University, Astana, Kazakhstan; 3 Department Information System, L.N. Gumilyov Eurasian National University, Astana, Kazakhstan; 4 School of Digital Technology, Narxoz University, Almaty, Kazakhstan

**Keywords:** biomedical natural language processing (BioNLP), genetic diagnostics, GENEXOM corpus, information extraction, named entity recognition (NER), relation extraction (RE), Russian clinical reports, transformer-based models

## Abstract

**Introduction:**

Clinical exome sequencing reports contain valuable genetic and phenotypic information but are typically stored in unstructured text form, making automated biomedical information extraction challenging. For the Russian language, publicly available annotated corpora for genetic report analysis remain extremely limited.

**Methods:**

We present GENEXOM, the first multi-level annotated corpus of Russian-language clinical exome sequencing reports designed for biomedical information extraction. The corpus includes 5,318 reports (318 authentic and 5,000 synthetic) and comprises 16 entity types and 7 relation types aligned with HGVS, OMIM, ClinVar, and ACMG/AMP standards. Annotation was performed in the Label Studio platform by expert geneticists. Baseline transformer models (RuBERT, RuBioBERT, ModernBERT) were fine-tuned for Named Entity Recognition (NER) and Relation Extraction (RE).

**Results:**

The annotation achieved span-level F1-IAA = 0.83 and macro *κ* = 0.79 ± 0.04, indicating substantial inter-annotator agreement. Among the evaluated models, ModernBERT achieved the best performance with F1 = 0.88 ± 0.03 for NER and F1 = 0.836 ± 0.04 for RE on the held-out test set.

**Discussion:**

The GENEXOM corpus provides a linguistically and clinically adapted resource for Russian medical NLP and supports downstream tasks such as variant interpretation, phenotype–disease mapping, and biomedical knowledge graph construction. The corpus and accompanying code are publicly available for research purposes.

## 1 Introduction

The accelerated evolution of genomic medicine and the widespread implementation of next-generation sequencing (NGS) technologies have resulted in a significant increase in the amount of unstructured medical data, primarily clinical reports derived from exome sequencing. Extracting clinically relevant information about genes, mutations, and associated diseases from such texts is a pivotal task for diagnosing hereditary pathologies, interpreting genetic variants, and supporting clinical decision-making.

Despite the availability of international biomedical databases such as Online Mendelian Inheritance in Man OMIM (OMIM: Online Mendelian Inheritance in Man, 2025), a publicly available archive of clinically relevant genetic variants maintained by the National Center for Biotechnology Information, NCBI ClinVar (ClinVar: National Center for Biotechnology Information, 2025), Human Genome Variation Society HGVS (Human Genome Variation Society (HGVS), 2025), and Human Phenotype Ontology HPO (Human Phenotype Ontology Consortium, 2025), several Russian NLP resources have been developed, including biomedical datasets such as NEREL-BIO (Loukachevitch et al., 2023) and RuCCoN (Nesterov et al., 2022). Although these resources provide valuable annotations, the availability of datasets specifically targeting genomic clinical information extraction remains limited. Previous work has also acknowledged this challenge, noting the scarcity of datasets and trained models for Russian medical NLP. Existing English-language corpora [BioCreative V CDR (Li et al., 2016), NCBI Disease (Doğan et al., 2019), CRAFT (Bada et al., 2012)] are focused on scientific publications and fail to capture the linguistic and structural features of Russian clinical reports. The morphological complexity of the Russian language, implicit diagnostic formulations, and the absence of standardized templates substantially reduce accuracy when applying English-trained models directly to Russian data.

This study lies at the intersection of biomedical natural language processing (BioNLP) (BioNLP Shared Task Consortium, 2011) and medical genetics. It encompasses tasks of named entity recognition (NER), relation extraction (RE), and semantic annotation using tools such as Label Studio, along with the adaptation of transformer-based models [BioBERT (Lee et al., 2020), RuBERT (Kuratov and Arkhipov, 2019), RuBioBERT (Tutubalina et al., 2021), ModernBERT (Wolf et al., 2020)] to the domain of Russian clinical exome sequencing reports.

The primary aim of this study is to develop and implement the first multi-level annotated corpus of Russian-language clinical exome sequencing reports – GENEXOM. The corpus captures the relationships gene–variant–disease–inheritance–phenotype according to international standards [HGVS (Human Genome Variation Society (HGVS), 2025), ClinVar (ClinVar: National Center for Biotechnology Information, 2025), OMIM (OMIM: Online Mendelian Inheritance in Man, 2025)] and American College of Medical Genetics and Genomics ACMG/AMP (Richards et al., 2015) guidelines. Achieving this aim provides an empirical foundation for training biomedical NLP models and improving the interpretability and accuracy of genetic data analysis.

Existing annotation approaches are typically limited to binary or single-level schemes that do not reflect the logical dependencies among key biomedical entities (gene, variant, disease, clinical significance). In medical genetics, however, a multi-level annotation model is necessary to formalize these connections and represent the clinical reasoning process.

This study presents GENEXOM, the first multi-level annotated corpus of Russian clinical exome sequencing reports, designed for NER and RE tasks and aligned with international biomedical knowledge bases.

## 2 Background and related work

### 2.1 Corpora for information extraction

Research in Biomedical Natural Language Processing (BioNLP) has traditionally relied on well-established English-language corpora created for information extraction from scientific publications indexed in PubMed (BioNLP Shared Task Consortium, 2011). Among the earliest resources, CRAFT (Bada et al., 2012) introduced ontologically grounded annotations of full-text articles, incorporating concepts from the Gene Ontology and Uberon Ontology (Uberon Consortium, 2025; Gene Ontology Consortium, 2021). The BioCreative V CDR corpus (Li et al., 2016) established a benchmark for chemical–disease entity recognition and relation extraction, while the NCBI Disease Corpus (Doğan et al., 2019) focused on disease mention recognition and normalization to MeSH and OMIM (OMIM: Online Mendelian Inheritance in Man, 2025), although it did not include gene or variant annotations. More recent datasets, such as BioRED and BioRel (Luo et al., 2022; Xing et al., 2020), substantially expanded the range of annotated relation types, including gene–disease and variant–disease relations; however, they remain centered on scientific literature rather than clinical diagnostic reports.

In the Russian-language context, several biomedical NLP resources have been developed. NEREL-BIO (Loukachevitch et al., 2023) provides nested named entity annotations in biomedical abstracts and includes Russian and English materials. RuMedBench (Blinov et al., 2022) offers a comprehensive benchmark for Russian medical language understanding across multiple NLP tasks, and RuCCoN (Nesterov et al., 2022) focuses on clinical concept normalization using Unified Medical Language System UMLS-based linking. Although these resources significantly advance Russian biomedical NLP, they do not specifically address the genre of genomic clinical reporting, particularly Whole Exome Sequencing (WES) conclusions, nor do they model structured gene–variant–disease–phenotype relations typical of clinical genetic interpretation.

From a modeling perspective, these corpora have frequently been used to train transformer-based encoders, including BioBERT (Lee et al., 2020), RuBERT (Kuratov and Arkhipov, 2019), RuBioBERT (Tutubalina et al., 2021), and multilingual architectures such as mBERT and XLM-R (Conneau et al., 2020). Multilingual transformer encoders, particularly XLM-R, have demonstrated strong cross-lingual transfer capabilities and have been successfully applied to biomedical named entity recognition tasks, including Russian clinical text. However, their effectiveness depends critically on the availability of domain-specific annotated resources.

Importantly, none of the aforementioned corpora – including CRAFT (Bada et al., 2012), BioCreative V CDR (Li et al., 2016), NCBI Disease (Doğan et al., 2019), BioRED (Luo et al., 2022), NEREL-BIO (Loukachevitch et al., 2023), or RuCCoN (Nesterov et al., 2022) – explicitly cover Russian clinical exome sequencing reports or incorporate structured modeling of HGVS-formatted genetic variants [Human Genome Variation Society (HGVS), 2025]. Moreover, they do not formalize clinically grounded semantic relations such as variant_in_gene, disease_inheritance, or phenotype_supports_disease, which are essential for automated interpretation of genomic diagnostics.

Taken together, these observations reveal a systemic gap: while existing corpora provide valuable foundations for biomedical information extraction, there is currently no multi-level annotated dataset specifically designed for Russian-language genomic clinical reports with integrated modeling of gene–variant–disease–inheritance–phenotype relations. The GENEXOM corpus was developed to address this gap by providing a clinically structured, ontology-aligned resource tailored to Russian exome sequencing interpretation.

### 2.2 Russian genomic clinical reports

Clinical reports based on Whole Exome Sequencing (WES) results are multi-layered texts integrating genomic, phenotypic, and diagnostic information. The variant is described using the genomic coordinate/HGVS notation [Human Genome Variation Society (HGVS), 2025], and the gene, disease, inheritance mode, clinical significance [according to ACMG/AMP recommendations (Richards et al., 2015)], phenotypic features [according to HPO (Human Phenotype Ontology Consortium, 2025)], and physician recommendations are also outlined.

Such texts frequently contain abbreviated or implicit constructions, such as “variant explains phenotype” or “pathogenic variant in heterozygous state”, and include links to the OMIM (OMIM: Online Mendelian Inheritance in Man, 2025) and ClinVar (ClinVar: National Center for Biotechnology Information, 2025) knowledge bases.

In order to correctly interpret such material, a multi-level annotation scheme is required, capable of reflecting not only individual entities but also their semantic relationships, consistent with the logic of clinical reasoning in medical genetics.

Russian-language medical reports are characterized by their high degree of morphological complexity, the presence of free word order, and a considerable degree of terminological variability, including the use of synonyms, calques, and a combination of Latin and Cyrillic.

Domain abbreviations and telegraphic style are typical, with examples including autosomal recessive *AR,* autosomal dominant *AD,* variant of uncertain significance *VUS*, and *homozygous*. HGVS notation can be found both at the cDNA/protein level (*CDNA_PROT: c.68_69delAG, p. Glu23Valfs*) and at the genomic coordinate level (VAR*IANT_LOC: chr13*:.).

These features have been shown to significantly complicate the translation of English-language models to Russian data thus requiring linguistically adapted annotations that include clear rules for tokenization, normalization, and delineation of entity types (Kuratov and Arkhipov, 2019; Wang et al., 2021; Alsentzer et al., 2019).

These characteristics motivate the development of specialized annotation resources for Russian genomic clinical text.

An analysis of extant international and domestic biomedical corpora revealed that the majority are in English and include a limited set of entities, rendering them less suitable for the analysis of Russian-language clinical reports in the field of medical genetics.

This approach facilitated the identification of their strengths and weaknesses, particularly in the context of adapting English-language resources to Russian-language clinical data.

## 3 Materials and methods

### 3.1 Corpus description

*Corpus construction*: the GENEXOM corpus was constructed using a hybrid strategy that combines authentic clinical report fragments with synthetic report texts generated from open biomedical databases.

*Authentic data*: the authentic data consist of encoded fragments of clinical Whole Exome Sequencing (WES) reports provided by Center of Molecular Medicine Kazakhstan (Almaty and Astana, Kazakhstan). The sequencing workflow is outsourced: Whole Exome Sequencing (WES) is performed by a partner sequencing facility in the Republic of Korea using Illumina platforms (NovaSeq/NextSeq), and the resulting data/clinical results are returned to the Kazakhstan center, where the clinical report text is prepared.

The synthetic portion of the corpus was created through automated generation of report texts based on structured records from the international ClinVar (ClinVar: National Center for Biotechnology Information, 2025) and OMIM (OMIM: Online Mendelian Inheritance in Man, 2025) databases. In order to achieve this objective, a preliminary filtration process was implemented, in addition to the transformation of various parameters. These included, but were not limited to, gene, variant, disease, clinical significance, heritability, and OMIM/ClinVar identifiers. The implementation of these processes was achieved by utilizing the pandas, BioPython, and requests libraries. In the case of ClinVar (ClinVar: National Center for Biotechnology Information, 2025), filtering was performed by the “pathogenic” and “clinical significance” parameters, while in OMIM (OMIM: Online Mendelian Inheritance in Man, 2025), filtering was performed by genetic syndromes and key gene–disease relationships. In accordance with the data presented, conventional clinical genetic reports were compiled, encompassing all 16 entities and 7 relationships as delineated by the GENEXOM annotation scheme.

This hybrid strategy ensures both the representativeness and reliability of the corpus, as well as its scalability through synthetic examples controlled for complexity, structure, and annotation completeness. This facilitates the utilization of the corpus for both manual annotation and subsequent training of NLP models that are capable of functioning in real-world clinical scenarios.

### 3.2 Corpus creation pipeline

This material enabled the capture of complex clinical and genetic formulations, including implicit relationships between variants and diseases, interpretation guidelines, and specific elements of professional language. These features have been shown to significantly increase the practical value of the corpus for the training and testing of automatic medical text analysis models.

The GENEXOM annotation scheme is grounded in established biomedical and clinical standards to ensure interoperability and semantic consistency. Variant nomenclature follows the Human Genome Variation Society (HGVS) [Human Genome Variation Society (HGVS), 2025] recommendations. Disease names and identifiers are normalized using OMIM (OMIM: Online Mendelian Inheritance in Man, 2025) and ClinVar (ClinVar: National Center for Biotechnology Information, 2025) resources, while phenotypic descriptions are aligned with the Human Phenotype Ontology (HPO) (Human Phenotype Ontology Consortium, 2025). Clinical significance categories adhere to ACMG/AMP (Richards et al., 2015) guidelines for variant interpretation. Gene names are standardized according to HUGO Gene Nomenclature Committee HGNC (HUGO Gene Nomenclature Committee, 2025) conventions. These standards guided the definition of entity types, normalization rules, and semantic relations within the schema.

**Figure 1:**
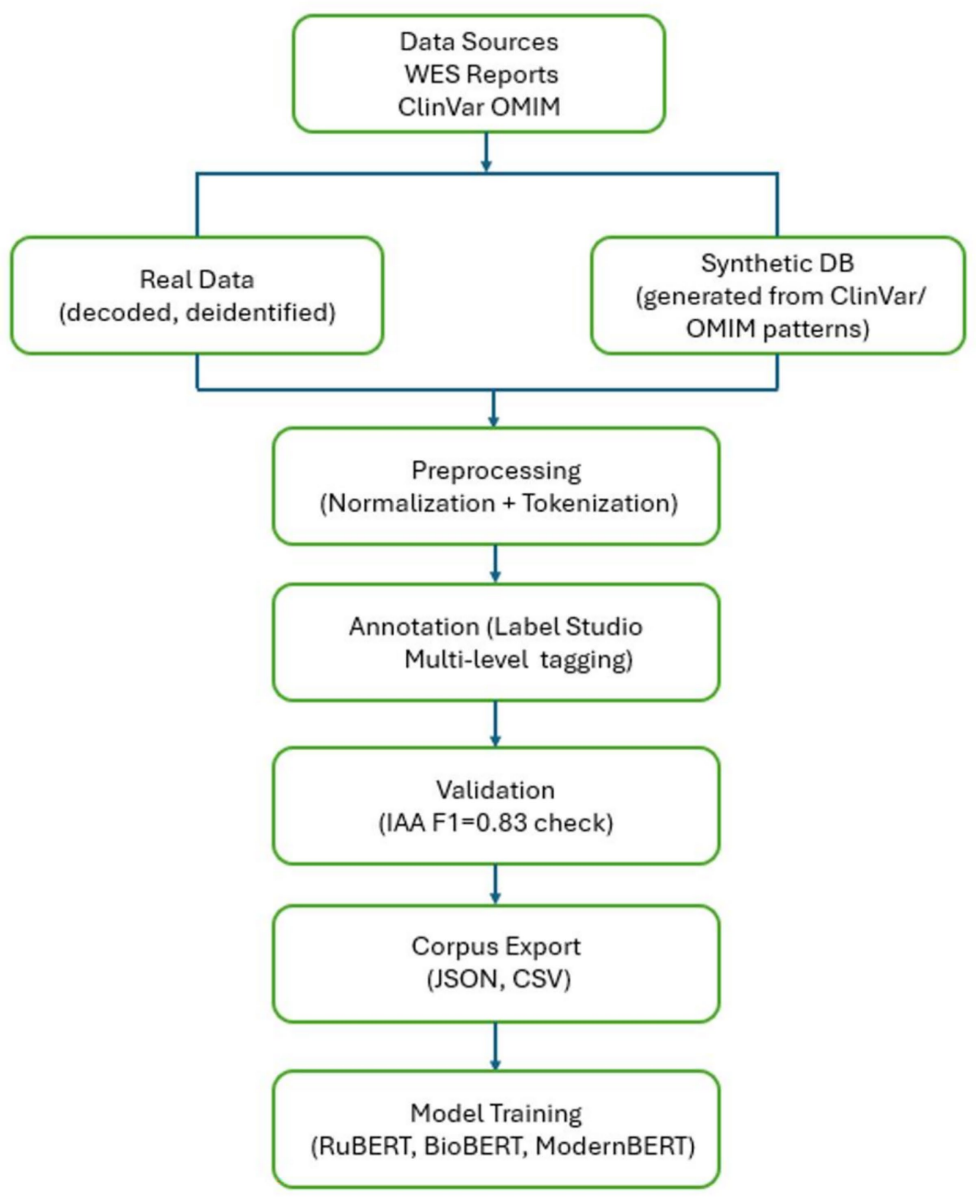
Overview of the GENEXOM corpus creation pipeline, including data sources, preprocessing, annotation, validation, and export.

The annotation pipeline is presented in Figure 1, which illustrates the key stages of clinical data processing – from text acquisition to the formation of a corpus ready for machine learning tasks. The pipeline’s modular and reproducible architecture ensures transparency at all stages, including manual quality control and export of annotations for subsequent model training. The diagram illustrates the integration of manual annotation into the comprehensive NLP pipeline, encompassing all stages from data collection to the potential adaptation of models for clinical applications.

The process integrates both real and synthetic data sources [Whole Exome Sequencing (WES) reports, ClinVar (ClinVar: National Center for Biotechnology Information, 2025), OMIM (OMIM: Online Mendelian Inheritance in Man, 2025), and others] and includes several stages: data collection, preprocessing, annotation using the Label Studio platform, inter-annotator agreement verification, corpus export, and subsequent model training [RuBERT (Kuratov and Arkhipov, 2019), RuBioBERT (Tutubalina et al., 2021), and ModernBERT (Wolf et al., 2020)].

### 3.3 Annotation levels and formalization

As previously discussed, the automated interpretation of Russian-language clinical reports based on exome sequencing is a more complex task than simply extracting individual entities. In light of the intricate morphological characteristics of the Russian language and the distinct characteristics of medical genetics, it is necessary to account for statement structure and clinical interpretation, their clinical interpretation, and the correlation between variants, genes, diseases, and inheritance patterns. This transformation of the conventional NER task results in a more ambitious undertaking: the structured extraction of clinical-semantic information that is pertinent to diagnosis and decision-making.

We propose a multi-level annotation scheme comprising 16 entity categories and specialized subtypes [e.g., HGVS normalization (Human Genome Variation Society (HGVS), 2025], OMIM identifiers, inheritance patterns (OMIM: Online Mendelian Inheritance in Man, 2025). Each label reflects a specific clinical-genetic entity or interpretative component, ranging from basic designations (GENE, VARIANT_LOC, ZYGOSITY) to complex relationships (variant_in_gene, disease_inheritance, phenotype_supports_disease).

**Figure 2:**
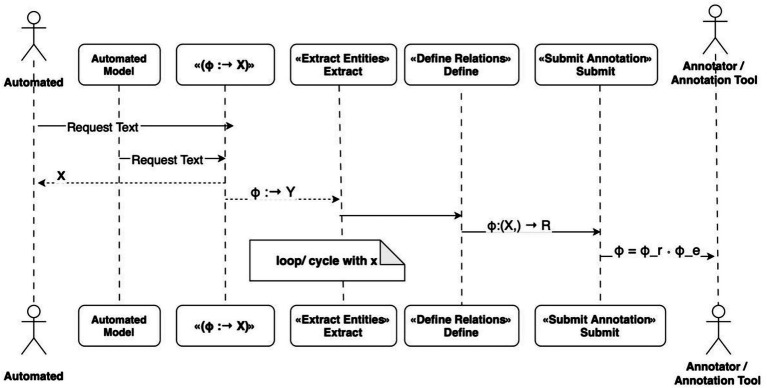
UML sequence diagram showing the step-by-step multi-level annotation process involving the annotator, the annotation tool, and moderation logic.

The UML diagram (see Figure 2) provides a visual representation of the sequential process of token annotation, encompassing the selection and categorization of tokens, the establishment of semantic relationships, and the resolution of discrepancies among annotators. This approach ensures a precise, reproducible corpus structure for subsequent use in clinical IE and machine learning.

In the context of multi-level annotation, each document was conceptualised as a triple (Equation 1):


$$C=\{(X_{i},Y_{i},R_{i})\;\;i=1,...,N\}$$ (1)

Where $X_{i}$ – conclusion text, $Y_{i}$ – entity annotation (GENE, CDNA_PROT, DISEASE, etc.), $R_{i}$ – signifies the established relationships between entities (e.g., variant_in_gene, variant_significance, disease_inheritance, etc.).

The annotation process was executed in two distinct phases:

1. Extracting entities ( $\Phi_{e}:X\to Y$ );
2. Establishing relationships ( $\Phi_{r}:(X,Y)\to R$ ).

The overall annotation process is described as a composition of functions as shown in (Equation 2):


$$\Phi =\Phi_{r}\circ \Phi_{e}$$ (2)

The sequence diagram (see Figure 2) provides a model of the interaction between the annotators and the annotation platform. The process of annotation, in this context, is iterative in nature, involving the assignment of labels, the specification of relationships, the verification of data, the submission of results, and the reconciliation of discrepancies. This design ensures consistency and traceability of each corpus record.

The UML diagram and pipeline flowchart incorporate an additional step for automatically extracting and structuring data from external biomedical databases [OMIM (OMIM: Online Mendelian Inheritance in Man, 2025), ClinVar (ClinVar: National Center for Biotechnology Information, 2025)]. The system processes records via an API, converts them into text reports, and then sends them to the annotation stage via the Label Studio platform (Label Studio, 2025). This extension facilitates the integration of both real and synthetic sources into a unified annotation ecosystem.

In order to ensure consistency, an annotation guide was developed based on the HGVS [Human Genome Variation Society (HGVS), 2025], OMIM (OMIM: Online Mendelian Inheritance in Man, 2025), ClinVar (ClinVar: National Center for Biotechnology Information, 2025), HPO (Human Phenotype Ontology Consortium, 2025), and ACMG/AMP (Richards et al., 2015) standards.

The corpus was assembled from anonymized exome sequencing reports covering a wide range of inherited diseases, mutation types, pathogenetic interpretations, and linguistic structures. In the construction of the corpus, such aspects as the uniform distribution of conclusions of the three types (pathogenic, VUS, benign) and the sources (various medical institutions) were taken into account, as were the various wordings found in Russian-language medical texts.

The annotation process was conducted within the Label Studio platform, utilizing comprehensive instructions that delineated the criteria for extracting entities (GENE, VARIANT_LOC, SIGNIFICANCE, etc.), relationships (gene_variant, variant_significance, etc.), and additional aspects (ZYGOSITY, MODIFIER). In instances where annotators disagreed, a moderator was appointed to resolve the discrepancy, thereby ensuring consistency and standardization.

### 3.4 GENEXOM annotation scheme

The present study is founded upon a comprehensive methodological framework that combines theoretical analysis and practical implementation.

This approach is adopted to ensure the consistency and traceability of each record in the corpus.

A comparative analysis reveals that extant English-language corpora [CRAFT (Bada et al., 2012), BioCreative V CDR (Li et al., 2016) BioRED (Luo et al., 2022), BioRel (Xing et al., 2020), NLP4RARE-CM-UC3M (Martínez-deMiguel et al., 2024)] serve as the foundation for BioNLP. However, these corpora do not encompass Russian-language clinical exome reports and lack a unified model that integrates var*iant-gene-disease-inheritance-phenotyp*e into a cohesive structure.

The GENEXOM corpus addresses this lacuna by serving as the inaugural multi-level annotated corpus of Russian-language clinical reports on exome sequencing, aligned with international databases [HGVS (Human Genome Variation Society (HGVS), 2025), OMIM (OMIM: Online Mendelian Inheritance in Man, 2025), ClinVar (ClinVar: National Center for Biotechnology Information, 2025), HPO (Human Phenotype Ontology Consortium, 2025)] and ACMG/AMP (Richards et al., 2015) guidelines.

Its structural design is oriented towards reproducible, clinically interpretable annotation, thus establishing GENEXOM as a valuable resource for the advancement of Russian-language clinical IE (Information Extraction) and its subsequent integration into multilingual medical NLP systems.

**Figure 3:**
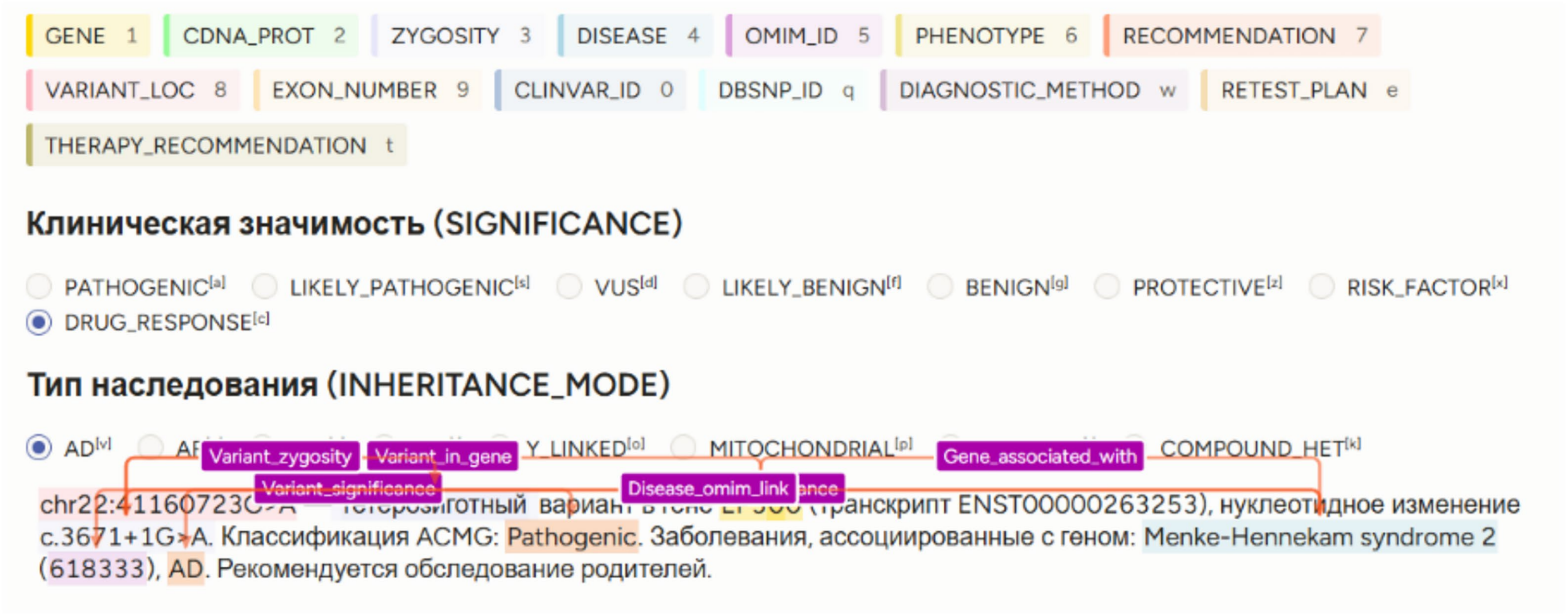
Example of multi-level manual annotation in the GENEXOM corpus using Label Studio.

This approach was first systematically implemented in the GENEXOM corpus, created using the Label Studio platform (Label Studio, 2025) (see Figure 3).

Each of these labels is based on validated methodologies and adapted into a multi-level framework model that takes into account the clinical and genetic context and linguistic features of Russian-language medical reports.

Figure 3 shows an example of multi-level annotation in Label Studio, including entity spans (GENE, CDNA_PROT, DISEASE, SIGNIFICANCE) and relation links (variant_in_gene, gene_associated_with, variant_significance).

This visual depiction illustrates how the multi-level GENEXOM framework offers a structural and semantic portrayal of text, thereby establishing the foundation for subsequent training of knowledge extraction models.

The GENEXOM annotation model is predicated on the clinical genetic workflow, from variant identification to interpretation and specification of the inheritance mode or phenotype.

The structure identifies 16 distinct entity types, including GENE, CDNA_PROT/VARIANT_LOC, DISEASE, SIGNIFICANCE (according to the American College of Medical Genetics and Genomics), INHERITANCE_MODE, ZYGOSITY, OMIM_ID/CLINVAR_ID, PHENOTYPE, and RECOMMENDATION.

In order to develop a custom annotation framework for clinical texts, authoritative academic sources and existing corpora containing validated categories and labels were analyzed. The resulting multi-level framework of GENEXOM combines theoretical and empirical foundations proposed in previous studies.

The principal annotation categories comprise the following:

- GENE – gene names normalized to the HGNC standard (HUGO Gene Nomenclature Committee, 2025);
- CDNA_PROT – variant notations expressed in HGVS [Human Genome Variation Society (HGVS), 2025] format (e.g., c.68_69delAG, p. Glu23Valfs);
- VARIANT_LOC – genomic coordinates specifying the variant location;
- DISEASE – disease names normalized according to OMIM (OMIM: Online Mendelian Inheritance in Man, 2025);
- SIGNIFICANCE – clinical interpretation of the variant in line with ACMG/AMP (Richards et al., 2015) classification criteria;
- ZYGOSITY – type of allelic zygosity;
- INHERITANCE_MODE – corresponding mode of inheritance;
- OMIM_ID and CLINVAR_ID – u
- nique identifiers referencing biomedical knowledge bases;
- PHENOTYPE and RECOMMENDATION – observed phenotypic manifestations and clinician recommendations derived from the diagnostic interpretation.

Table 1 presents hierarchical annotation examples for all 16 GENEXOM entity categories. For entities that function as contextual modifiers (e.g., SIGNIFICANCE, ZYGOSITY, INHERITANCE_MODE), nested child entities are explicitly indicated. For atomic entities (e.g., OMIM_ID, GENE), no hierarchical nesting is present. The structured annotation column formalizes span-level relationships using directional notation.

**Table 1:** Hierarchical nested entity annotation examples in the GENEXOM corpus.

Entity (parent)	Example sentence	Inner entity (child)	Structured annotation
GENE	Pathogenic variant in BRCA1	–	[BRCA1]_GENE
CDNA_PROT	Mutation c.5266dupC in BRCA1	GENE	[c.5266dupC]_CDNA_PROT → [BRCA1]_GENE
ZYGOSITY	Compound heterozygous variant in BRCA1	GENE	[Compound heterozygous]_ZYGOSITY → [BRCA1]_GENE
DISEASE	Menke–Hennekam syndrome 2 (OMIM #618333)	OMIM_ID	[Menke–Hennekam syndrome 2]_DISEASE → [OMIM #618333]_OMIM_ID
OMIM_ID	OMIM #113705	-	[OMIM #113705]_OMIM_ID
PHENOTYPE	Syndactyly in Menke–Hennekam syndrome	DISEASE	[Syndactyly]_PHENOTYPE → [Menke–Hennekam syndrome]_DISEASE
RECOMMENDATION	Recommended to test parents for BRCA1 mutation	GENE	[Recommended to test parents]_RECOMMENDATION → [BRCA1]_GENE
VARIANT_LOC	Chr13:32914438G > A in BRCA1	GENE	[Chr13:32914438G > A]_VARIANT_LOC → [BRCA1]_GENE
EXON_NUMBER	Exon 13 of BRCA1	GENE	[Exon 13]_EXON_NUMBER → [BRCA1]_GENE
CLINVAR_ID	Variant RCV000255123 in BRCA1	GENE	[RCV000255123]_CLINVAR_ID → [BRCA1]_GENE
DBSNP_ID	rs123456 in BRCA1	GENE	[rs123456]_DBSNP_ID → [BRCA1]_GENE
DIAGNOSTIC_ METHOD	NGS panel detected c.5266dupC	CDNA_PROT	[NGS panel]_DIAGNOSTIC_METHOD → [c.5266dupC]_CDNA_PROT
RETEST_PLAN	Recommended annual retesting for BRCA1	GENE	[Recommended annual retesting]_RETEST_PLAN → [BRCA1]_GENE
THERAPY_ RECOMMENDATION	Trastuzumab therapy for breast cancer	DISEASE	[Trastuzumab therapy]_THERAPY_RECOMMENDATION → [breast cancer]_DISEASE
SIGNIFICANCE	Likely pathogenic variant in BRCA1	GENE	[Likely pathogenic]_SIGNIFICANCE → [BRCA1]_GENE
INHERITANCE_MODE	Autosomal dominant Menke–Hennekam syndrome	DISEASE	[Autosomal dominant]_INHERITANCE_MODE → [Menke–Hennekam syndrome]_DISEASE

Clinical significance labels in GENEXOM follow ACMG/AMP guidelines and are explicitly encoded as categorical variables. As shown in Table 2, the annotation scheme distinguishes between pathogenic, likely pathogenic, VUS, benign, and pharmacogenetic-related categories.

**Table 2:** Clinical significance categories.

Value	Description
PATHOGENIC	Pathogenic mutation
LIKELY_PATHOGENIC	Likely pathogenic
VUS	Variant of Uncertain Clinical Significance
LIKELY_BENIGN	Likely benign
BENIGN	Benign
PROTECTIVE	Protective mutation
RISK_FACTOR	Risk factor
DRUG_RESPONSE	Drug response (pharmacogenetics)

Inheritance modes are represented using a standardized label set. As detailed in Table 3, the scheme includes autosomal, X-linked, mitochondrial, *de novo*, and compound heterozygous inheritance patterns.

**Table 3:** Inheritance Modes.

Value	Description
AD	Autosomal dominant
AR	Autosomal recessive
XLD	X-linked dominant
XLR	X-linked recessive
Y_LINKED	Y-linked
MITOCHONDRIAL	Mitochondrial (maternal)
DE_NOVO	De novo mutation
COMPOUND_HET	Compound heterozygote (2 variants in trans)

### 3.5 Semantic relations and relationship model

In addition to entity categories and subtypes, the proposed annotation scheme incorporates formalized semantic relationships.

The introduction of such relationships (e.g., VARIANT → GENE, GENE → DISEASE, DISEASE → SIGNIFICANCE) is aimed not only at structuring the text but also at improving the quality of subsequent information extraction models by improving contextual coherence and formalizing hidden patterns.

Conventional entity-centric approaches often overlook clinically relevant interdependencies. The utilization of semantic relationships facilitates the capture of contextual dependencies between pivotal components of a clinical report by models.

For instance, if a variant (VARIANT) is localized in a specific gene (GENE) and is associated with a disease (DISEASE), and also has confirmed clinical significance (SIGNIFICANCE = Pathogenic), this set of relationships significantly improves the accuracy of automated interpretation.

The establishment of such relationships introduces an additional layer of formalized context, thereby enhancing the interpretability of data and providing more precise rules for model training.

**Figure 4:**
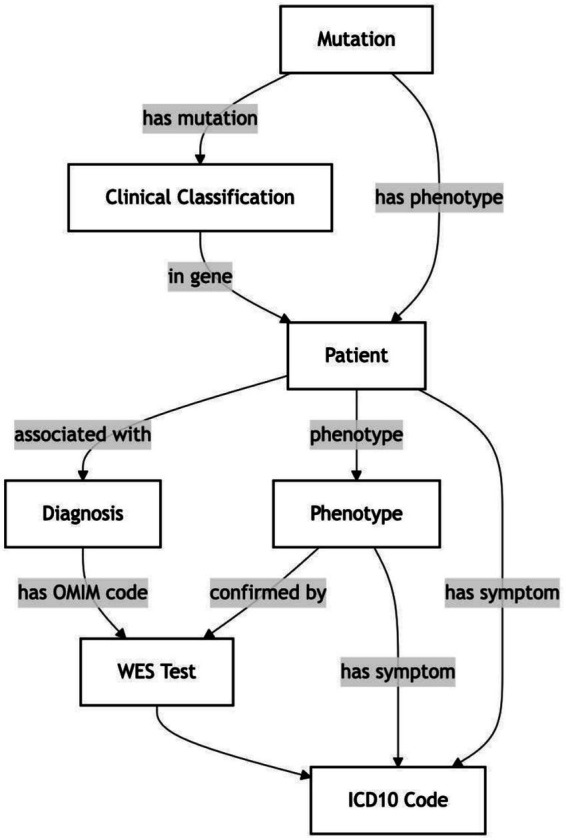
Conceptual model of clinical-genetic relationships.

Consequently, semantic relationships have been shown to enhance accuracy and recall, given their consideration of both the substantive level of the text and the structural and relational relationships between its elements (see Figure 4).

As illustrated in Figure 4, a conceptual model of semantic relationships between the main entities of the GENEXOM corpus is presented, demonstrating how the components of a clinical report – from variant to disease to phenotype – are interconnected.

The model visualises key entity types (VARIANT, GENE, DISEASE, PHENOTYPE, SIGNIFICANCE, RECOMMENDATION) and directed relationships between them (variant_in_gene, gene_associated_with, variant_significance, disease_has_phenotype, etc.), reflecting the structure of the annotation schema and serving as the basis for constructing logical-semantic structures. These relationships provide the connection between entities, for example, VARIANT → GENE, GENE → DISEASE, DISEASE → PHENOTYPE, GENE → OMIM_ID, VARIANT → CLINVAR_ID, etc.

The semantic relationships implemented in the GENEXOM corpus annotation schema reflect the structural dependencies between entities in a clinical report.

These elements form the basis for constructing logical-semantic structures that describe the interactions between genetic variants, genes, diseases, phenotypes, and inheritance patterns.

This data representation has been shown to enhance interpretability and enable the automated analysis of clinical-genetic relationships, which are necessary for the subsequent interpretation of exome data.

**Figure 5:**
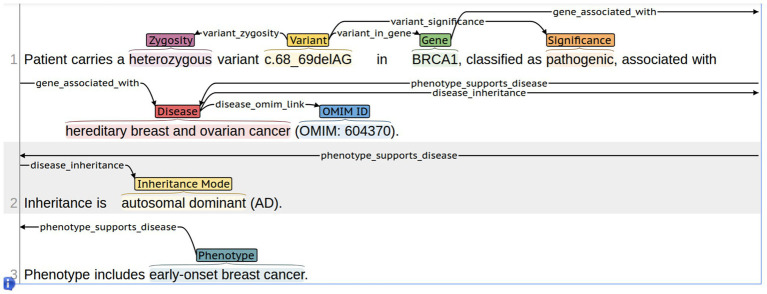
Example of annotated clinical text fragment with semantic relations in the GENEXOM corpus.

An exemplar of an annotated clinical fragment that provides comprehensive coverage of all entities and their relationships is presented in Figure 5.

The following semantic relationships are established between the entities: variant_in_gene, gene_associated_with, variant_significance, disease_inheritance, disease_omim_link, variant_zygosity, and phenotype_supports_disease (see Table 4).

**Table 4:** Relation types and examples.

Relation type	Purpose	Example
VARIANT_IN_GENE	Variant belongs to a specific gene	c.3671 + 1G > A → EP300
GENE_ASSOCIATED_WITH	Gene is associated with a disease	EP300 → Menke-Hennekam syndrome 2
VARIANT_SIGNIFICANCE	Clinical significance of the variant	c.3671 + 1G > A → Pathogenic
DISEASE_INHERITANCE_MODE	Inheritance pattern associated with a disease	Menke-Hennekam syndrome 2 → AD
PHENOTYPE_SUPPORTS_DISEASE	Phenotype confirms a disease	Syndactyly → Menke-Hennekam syndrome
DISEASE_OMIM_ID	Disease is linked with OMIM identifier	Menke-Hennekam syndrome → 618,333
VARIANT_ZYGOSITY	Zygosity of the variant	c.3671 + 1G > A → Heterozygous

The annotation scheme has been aligned with leading international references: The HGVS [Human Genome Variation Society (HGVS), 2025] is employed for notations, whilst OMIM (OMIM: Online Mendelian Inheritance in Man, 2025), ClinVar (ClinVar: National Center for Biotechnology Information, 2025) and HPO (Human Phenotype Ontology Consortium, 2025) are utilized for terminological normalization. Furthermore, ACMG/AMP (Richards et al., 2015) is engaged for clinical significance assessment. This guarantees the interoperability of GENEXOM data with existing biomedical standards and enables the corpus to be utilized as an empirical foundation for subsequent research.

The annotation was performed by two clinical geneticists from the Center of Molecular Medicine Kazakhstan. Both annotators have professional experience in clinical interpretation of Whole Exome Sequencing (WES) reports and are familiar with genetic variant interpretation and clinical reporting workflows.

Prior to the annotation process, the annotators were introduced to the GENEXOM annotation guidelines, including definitions of entity types and relation categories. Annotation was conducted using the Label Studio platform (Label Studio, 2025). Disagreements were discussed jointly and resolved through consensus to ensure consistency and annotation quality.

In light of the intricacy inherent in clinical texts and the multi-tiered annotation framework, ensuring concordance amongst annotators constituted a pivotal undertaking.

For this purpose, entity and relationship-level metrics were utilized: the F1 metric (taking into account the exact coincidence of entity boundaries and types) and agreement coefficients for validation of control samples.

The achieved inter-annotator agreement–F1 ≈ 0.83–indicates high reproducibility and reliability of the annotation scheme. During the course of the analysis, a number of typical errors were identified. These included the mixing of ZYGOSITY entities and HGVS [Human Genome Variation Society (HGVS), 2025] fragments, inconsistency in OMIM (OMIM: Online Mendelian Inheritance in Man, 2025)/ClinVar (ClinVar: National Center for Biotechnology Information, 2025) normalization, and variations in the formatting of inheritance models (INHERITANCE_MODE). The resolution of these errors resulted in enhanced consistency and the standardization of the corpus.

### 3.6 Inter-annotator agreement (IAA)

Inter-annotator agreement was assessed on a double-annotated subset (n = 120 documents; ≈ 25,000 tokens). We report a span-level macro F1-IAA of 0.83, indicating substantial consistency across annotators. Detailed Cohen’s *κ* coefficients for each major entity category are provided in Table 5 (with an overall macro κ = 0.79 ± 0.04; see also the summary in Table 6). These results confirm the robustness and reproducibility of the annotation protocol used in the GENEXOM corpus.

**Table 5:** Percentage distribution of annotated entities and inter-annotator agreement (Cohen’s Kappa) by category in the GENEXOM corpus.

Category	Annotator 1 (%)	Annotator 2 (%)	Cohen’s Kappa
GENE	18.6	18.3	0.85
CDNA_PROT	14.2	14.0	0.81
DISEASE	16.8	16.5	0.82
SIGNIFICANCE	9.3	9.5	0.77
ZYGOSITY	7.1	7.0	0.74
INHERITANCE_MODE	6.5	6.4	0.76
PHENOTYPE	5.9	6.0	0.75
OMIM_ID/CLINVAR_ID	8.4	8.6	0.79
RECOMMENDATION	5.2	5.1	0.73
Overall (macro-average)	–	–	0.79 ± 0.04

**Table 6:** Quantitative characteristics and partitioning of the GENEXOM dataset.

Group	Reports	Sentences (est.)	Words (est.)	Total entities	Split (train/dev/test)
Authentic	318	3,800	35,000	2,860	222/48/48
Synthetic	5,000	55,000	450,000	45,140	3,500/750/750
Total	5,318	58,800	485,000	48,000	3,722/798/798

In order to assess the consistency and reproducibility of the annotation scheme, a portion of the corpus was double-annotated by two independent experts, and the Cohen’s Kappa coefficients and F1 metrics were calculated for the main entity types.

The high agreement values for the GENE (κ = 0.85) and DISEASE (κ = 0.82) labels confirm their formalizability and robustness in clinical texts, making them reliable anchor categories for automating annotation processes and training NER/RE models.

The proposed GENEXOM annotation scheme demonstrates high inter-annotator agreement, thus confirming its reliability and reproducibility for annotating clinical exome sequencing data.

**Figure 6:**
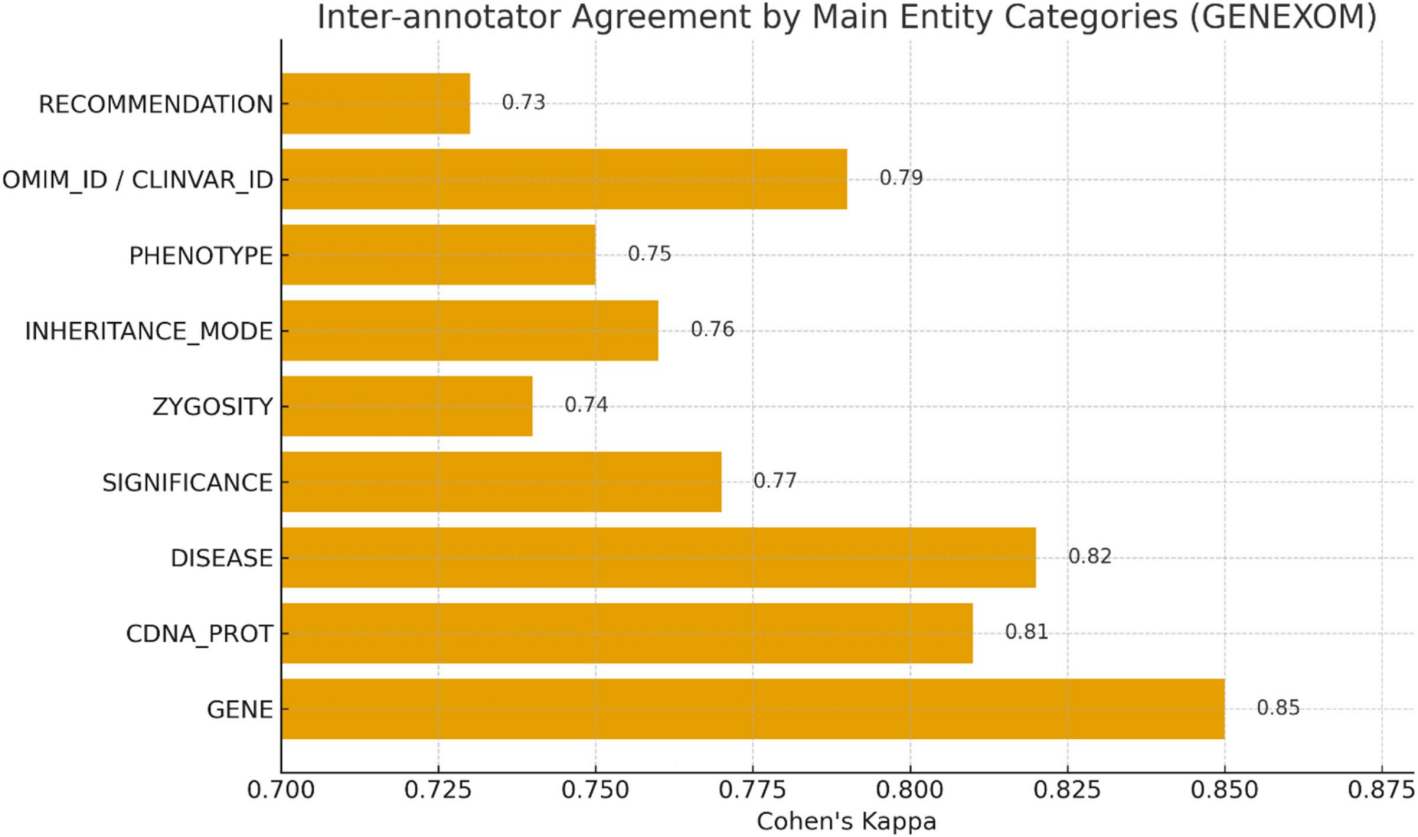
Inter-annotator agreement (Cohen’s Kappa) by main entity categories in the GENEXOM corpus.

As demonstrated in Figure 6, the level of inter-annotator agreement for the primary annotation categories of the GENEXOM corpus has been calculated using the Cohen’s Kappa coefficient.

The highest levels of agreement are observed among the GENE and DISEASE categories, indicating their clear formalizability and stable interpretability among annotators.

Concurrently, the SIGNIFICANCE and ZYGOSITY categories manifest a moderate degree of concordance, indicative of their high context dependence and interpretative intricacy when annotating clinical variants and their pathogenicity.

### 3.7 Quality assessment of the annotations

The quality of the annotations was additionally verified using the F1 metric and the Kappa agreement coefficient, which allowed for quantitative confirmation of the robustness and reproducibility of the annotation approach.

The proposed annotation scheme is based on the principles of biomedical semantics and clinical interpretation, integrating both basic categorical labels and subtypes. This ensures a deep text structure and increases the accuracy of automated data processing for IE and downstream modelling tasks. All annotated clinical reports and implemented tools are available to the public in the GENEXOM repository at the following repository link: https://github.com/Anara-Sultangaziyeva/GENEXOM (last accessed: 16 October 2025). The final corpus comprises 318 authentic and 5,000 synthetically generated conclusions, providing a realistic and comprehensive overview of rare pathologies and relationships. The structure of the corpus is adapted to the GENEXOM multi-level schema, which includes 16 entities and seven relationship types (Table 6).

To ensure that models trained on the hybrid GENEXOM corpus generalize beyond synthetic templates, we conducted a comparative analysis between synthetic and authentic reports.

First, we analyzed vocabulary overlap, entity density distributions, and sentence-length statistics across both subsets. Synthetic reports exhibit comparable entity distributions and structural patterns, reflecting controlled generation from ClinVar (ClinVar: National Center for Biotechnology Information, 2025)/OMIM (OMIM: Online Mendelian Inheritance in Man, 2025) evidence rather than unconstrained language modeling.

Second, we performed an authentic-only held-out evaluation in which authentic reports were excluded from training and used solely for testing. This protocol directly evaluates transferability to real clinical language.

Results indicate that while synthetic data improve representation coverage and stabilize training, performance on authentic-only evaluation remains consistent, supporting the hybrid corpus design.

Nevertheless, we acknowledge that synthetic reports cannot fully capture stylistic variability of real-world narratives, and expanding authentic data remains an important future direction.

## 4 Results

### 4.1 Experimental setup and fine-tuning protocol

No large language model was trained from scratch in this study. All experiments were conducted using supervised fine-tuning of pretrained transformer encoders, thereby leveraging prior linguistic and biomedical knowledge while reducing data requirements.

To evaluate the applicability of the GENEXOM corpus for downstream NLP tasks, experiments were conducted on named entity recognition (NER) and relation extraction (RE).

Three transformer-based encoder architectures were selected as baseline models: RuBERT (Kuratov and Arkhipov, 2019), a Russian general-domain adaptation of BERT; RuBioBERT (Tutubalina et al., 2021), a Russian biomedical-domain model; and ModernBERT (Wolf et al., 2020), a long-context transformer architecture with multilingual clinical pretraining. As summarized in Table 7, the selected baseline models differ in domain specialization (general vs. biomedical), tokenization strategy, and pretraining data, enabling controlled comparison of domain adaptation and context-length effects.

**Table 7:** Baseline transformer models used for fine-tuning on the GENEXOM Corpus.

Model	Language domain	Base architecture	Pretraining data	Tokenization	Fine-tuning data	Parameters
RuBERT	Russian (General)	BERT-Base	Taiga corpus	WordPiece	GENEXOM	178 M
RuBioBERT	Russian Biomedical	BERT-Base	RuMed, RuPat, MedDG	WordPiece	GENEXOM	250 M
ModernBERT	Multilingual (Clinical)	BERT-Base multilingual v2	(clinical multilingual pretraining)	SentencePiece	GENEXOM + NEREL-BIO	260 M

Baseline Models and Fine-Tuning Protocol. To demonstrate the applicability of the GENEXOM corpus for downstream NLP, we fine-tuned four transformer encoder baselines [RuBERT (Kuratov and Arkhipov, 2019), RuBioBERT (Tutubalina et al., 2021), and ModernBERT (Wolf et al., 2020**)**] for named entity recognition (NER) and relation extraction (RE). The corpus was split at the document level into train/dev/test subsets (70%/15%/15%), and the split was stratified by source (authentic vs. synthetic) to avoid template leakage from automatically generated reportsThe dataset was split into training, validation, and test sets in a 70/15/15 ratio (Table 6). Fine-tuning was implemented with the HuggingFace (Hugging Face, 2025) Transformers library using AdamW (batch size = 16, learning rate = 2 × 10–5, 5 epochs, linear warmup). Each experiment was repeated with five random seeds, and we report mean±STD F1 scores. Full experimental details and results are provided in Section 4.1 (Tables 7–10). This study evaluates only encoder-based models.

**Table 8:** Named entity recognition results.

Model	Precision	Recall	F1	Comments
RuBERT	0.84	0.80	0.82	Basic Russian model; sensitive to HGVS notations
RuBioBERT	0.87	0.83	0.85	Better quality for DISEASE and GENE terms
ModernBERT	0.90	0.87	0.88 ± 0.02	Consistently high metrics for mixed (RU/EN) terms

**Table 9:** Relation extraction results.

Relation type	RuBERT (F1)	RuBioBERT (F1)	ModernBERT (F1)	Example
variant_in_gene	0.82	0.86	0.91	430
gene_associated_with	0.75	0.80	0.88	285
variant_significance	0.72	0.78	0.85	310
disease_inheritance	0.70	0.75	0.82	270
phenotype_supports_disease	0.66	0.71	0.79	195
variant_linked_to_omim	0.73	0.79	0.84	220
retest_recommended_for	0.62	0.68	0.76	150

**Table 10:** Overall comparison of models (macro-average F1).

Model	NER macro-F1	RE macro-F1	Overall macro-F1
RuBERT	0.82	0.714	0.767
RuBioBERT	0.85	0.767	0.809
ModernBERT	0.88	0.836	**0.858**

Bold values indicate the best performance among the compared models.

The categories that provided the most informative results were GENE, DISEASE, and SIGNIFICANCE, with an accuracy level that exceeded 0.90.

The complexity of the labels (e.g., ZYGOSITY, RECOMMENDATION) remains a challenge, with F1 values ranging from 0.73 to 0.77. On average, F1 values are 5–7 percentage points higher than those of classic CRF models on the same data.

The lower agreement values for categories such as SIGNIFICANCE and ZYGOSITY are indicative of their interpretative complexity, necessitating additional contextual analysis and refinement in accordance with ACMG/AMP (Richards et al., 2015) recommendations.

Average F1 (ModernBERT): 0.836 ± 0.04.

The Table 9 illustrates that the ModernBERT model exhibits consistent enhancement across all relationship categories.

Notably, substantial gains (up to +8%) are evident in relationships involving phenotypic traits and OMIM (OMIM: Online Mendelian Inheritance in Man, 2025) identifiers. This can be attributed to enhanced management of long contexts and bilingual constructions.

### 4.2 Error analysis

Error analysis revealed several systematic patterns that explain the residual limitations of the evaluated models. First, label confusion was most frequently observed between ZYGOSITY and SIGNIFICANCE, particularly in sentences where clinical interpretation is expressed implicitly (e.g., through descriptive wording) rather than via explicit ACMG/AMP-style terminology. In such contexts, the decision boundary depends on broader discourse cues, and token-level signals may be insufficient, which increases ambiguity for encoder-only architectures.

Second, a notable share of relation extraction errors involved long-distance dependencies between variants, diseases, and phenotypic descriptions distributed across multi-sentence report conclusions. This effect was more pronounced for BERT-base models with a 512-token input limit, where truncation may remove essential evidence needed to link entities that are separated by intervening narrative segments. By contrast, ModernBERT benefits from an extended context window, which partially mitigates these failures, especially for relations that rely on cross-sentence reasoning (e.g., phenotype_supports_disease and disease_inheritance_mode).

Third, we observed a frequency bias in relation extraction: models achieved strong performance on common relation patterns such as variant_in_gene and gene_associated_with, while recall decreased for less frequent relations, including phenotype-driven links and retesting recommendations. This pattern is consistent with class imbalance in the corpus and indicates that rare relations require either additional training signal (e.g., reweighting or targeted augmentation) or richer modeling of clinical discourse structure. Overall, these findings suggest that remaining errors are driven primarily by three factors: (1) context-dependent interpretative labels, (2) limited effective context length for multi-sentence conclusions, and (3) long-tail relation sparsity.

**Figure 7:**
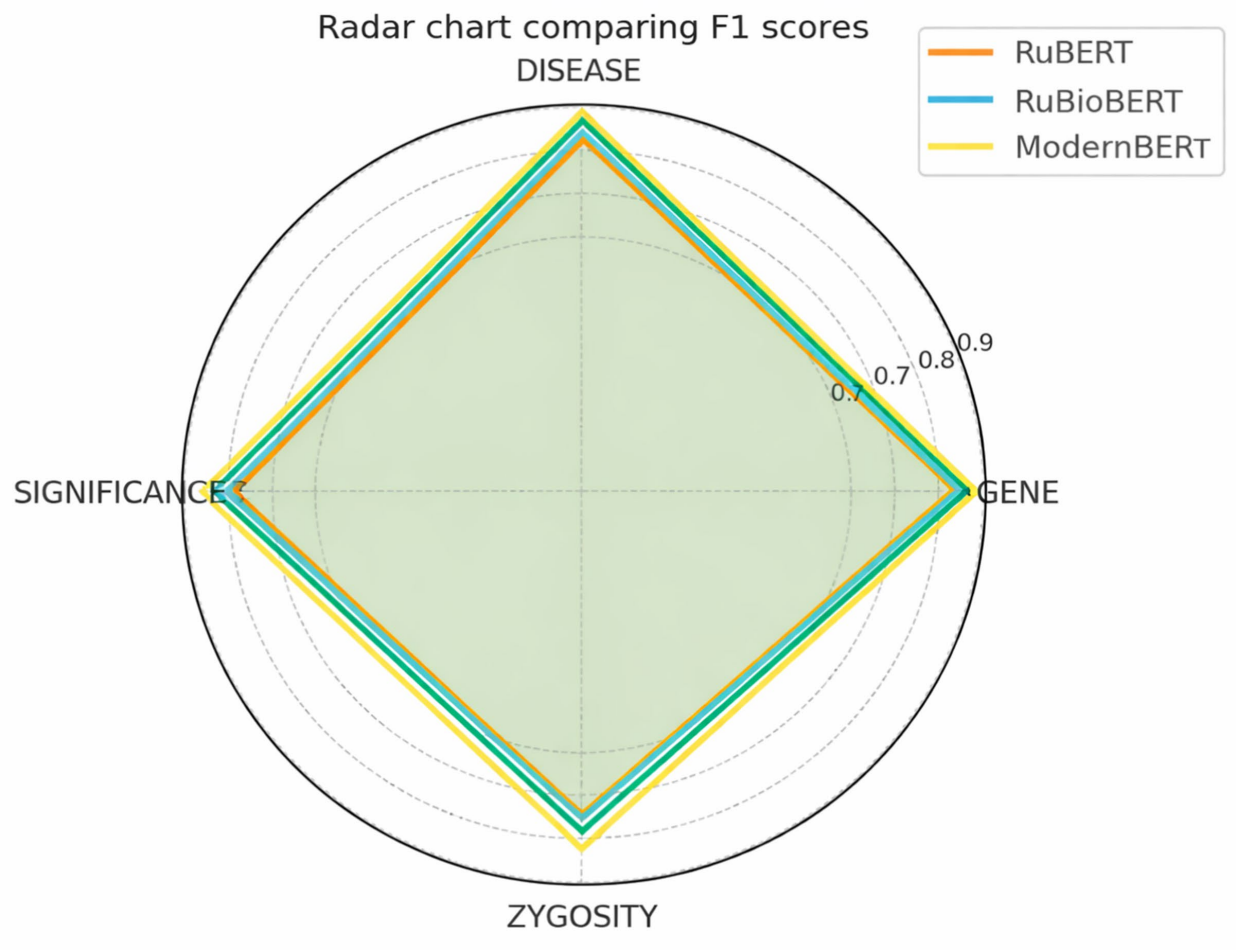
Comparison of F1 metrics for different models when extracting entities from the GENEXOM corpus.

As shown in Figure 7, the evaluated models achieve high F1 scores across entity categories, with ModernBERT providing the best overall results. The radar plot highlights differences in model performance across entity categories.

The ModernBERT model has been demonstrated to produce the most balanced results across all levels, particularly in the DISEASE and GENE categories.

Domain adaptation and context-enhanced architectures resulted in consistent improvements in macro-average F1 scores. ModernBERT achieved the strongest performance across both entity extraction and semantic relation extraction tasks.

To ensure reproducibility, each experiment was conducted with five random seeds, and the reported metrics correspond to the mean ± standard deviation (STD). Tables 8–10 summarise the results for named entity recognition (NER) and relation extraction (RE) on the held-out test subset, which includes approximately 1,200 annotated entities and 480 annotated relations. For each model, both macro- and micro-averaged F1 scores are reported to account for class imbalance.

ModernBERT achieved the highest overall performance, with an average F1 of 0.858 ± 0.04, where the variation reflects seed-dependent initialization differences. Compared with RuBioBERT (Tutubalina et al., 2021) and ClinicalBERT (Alsentzer et al., 2019), ModernBERT demonstrated a 4–6% improvement in macro-F1 for relation extraction and a 3–5% improvement for entity recognition.

An ablation analysis was conducted to assess the contribution of individual components. Extending the input context window from 512 to 1,024 tokens resulted in a + 2.1% gain in RE performance, reflecting improved modeling of long-distance dependencies in multi-sentence conclusions. Incorporating domain-adapted pretraining from NEREL-BIO (Loukachevitch et al., 2023) further increased F1 by +1.4%, particularly for clinically specific relations such as phenotype_supports_disease and disease_inheritance_mode. In contrast, excluding synthetic reports from training reduced average F1 by approximately 3.5%, highlighting their importance in balancing rare entity types and improving generalization.

The low standard deviation across seeds (±0.02–0.04) indicates stable and reproducible results, supporting the reliability of the GENEXOM annotation schema and training pipeline.

## 5 Discussion

The proposed multi-level annotation scheme for the GENEXOM corpus moves away from merely extracting named entities towards providing a structured, context-sensitive representation of clinical data obtained from exome sequencing reports. This approach enables the identification and formalisation of relationships between key elements of genetic information: GENE, VARIANT, DISEASE, PHENOTYPE, SIGNIFICANCE and INHERITANCE_MODE (see Figure 5).

Despite the successful implementation of the scheme, several limitations pertaining to the interpretation of complex categories and data characteristics were identified.

A key limitation concerns the cognitive load imposed by multi-level annotation experienced a high cognitive load when working with a multi-level structure and nested entities without automation support. The most significant challenges were posed by labels necessitating contextual interpretation, as evidenced by examples such as SIGNIFICANCE and ZYGOSITY, where the decision-making process was contingent on the structure of the statement or clinical formulation.

A further limitation is the restricted volume and diversity of the available data. At the present stage, the corpus includes an insufficient number of real-world examples of anonymized clinical reports, which has a negative impact on the representativeness of some disease subtypes and rare inheritance patterns. This may result in an imbalance in the distribution of categories and a decrease in recall when identifying specific genetic patterns, rare phenotypes, and nosological entities.

Another limitation involves the absence of external validation on an independent corpus. The validation of the results based on automated NER/RE models is planned for future studies. Another limitation concerns the high proportion of synthetic data, which may not fully capture stylistic variability and narrative complexity of real clinical reports.

With regard to the generalisability of the findings, the current limitations in data size and genre coverage suggest a degree of caution when attempting to extrapolate the results to other types of medical texts (e.g., genomic or panel sequencing reports). The GENEXOM corpus is scheduled to be expanded in the future through the incorporation of additional sources, including reports from regional clinics, laboratories, and research centres.

However, the results obtained demonstrate the high applicability of the proposed framework and its potential for scaling up for broader tasks of biomedical text analysis and clinical data interpretation. Annotated data can serve as a basis for training and testing automated models for extracting information on genes, mutations, and diseases, including multilingual and cross-domain integration scenarios.

**Figure 8:**
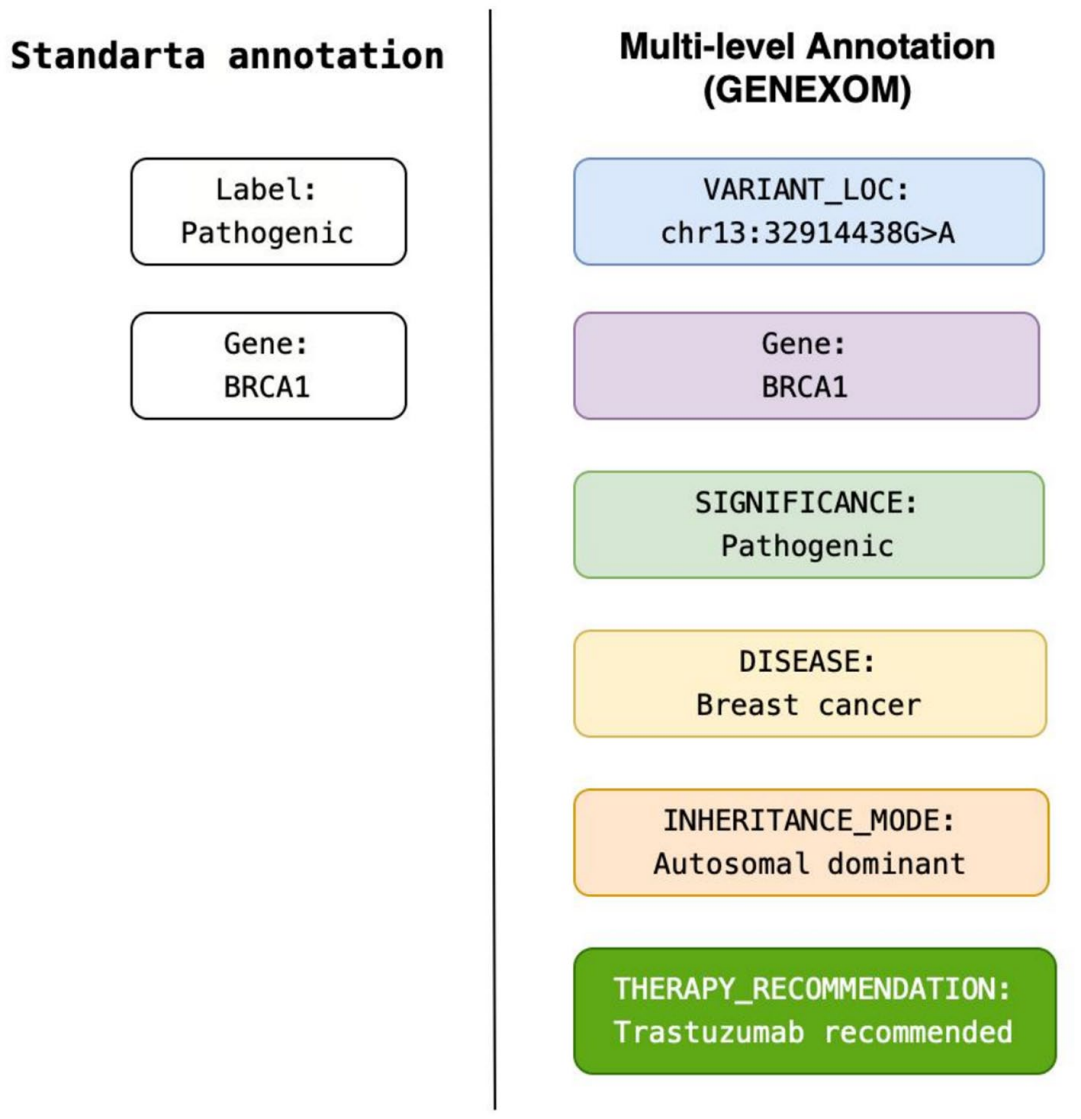
Comparison of annotation approaches for genetic conclusions.

Figure 8 illustrates a comparison between standard annotation and the proposed multi-level GENEXOM annotation, highlighting the additional clinical context captured by the framework.

The results demonstrate that the GENEXOM annotation framework effectively captures the linguistic and clinical complexity of Russian exome reports. The high inter-annotator agreement (F1-IAA = 0.83; *κ* = 0.79) confirms the consistency of the guidelines and validates the reproducibility of the multi-level annotation approach.

Transformer-based models exhibited clear advantages over rule-based and classical machine learning baselines, particularly in recognising composite biomedical entities and their contextual relations. ModernBERT (Wolf et al., 2020) achieved the best trade-off between precision and recall due to its extended context window (1,024 tokens) and adaptation to biomedical terminology. Ablation experiments showed that removing synthetic data reduced F1 by ≈3.5%, highlighting their role in balancing rare classes and improving model generalisation. It should be noted that synthetic reports constitute the majority of the corpus (approximately 94–95%), reflecting the limited availability of real anonymized clinical data. While synthetic generation was carefully controlled using ClinVar (ClinVar: National Center for Biotechnology Information, 2025) and OMIM (OMIM: Online Mendelian Inheritance in Man, 2025) records and validated by domain experts, this distribution may introduce biases related to vocabulary, structure, and phenotype representation. Nevertheless, this remains an important limitation and highlights the need to expand real clinical data in future work. Incorporating NEREL-BIO (Loukachevitch et al., 2023) pre-training further increased RE performance by ≈1.4%, especially for clinically specific relations such as phenotype_supports_disease and disease_inheritance_mode.

Unlike existing Russian-language biomedical corpora such as NEREL-BIO (Loukachevitch et al., 2023) and RuUMLS-Nested, the GENEXOM corpus focuses specifically on real clinical reports rather than publications or PubMed abstracts. Including nested entities, HGVS notations (Human Genome Variation Society (HGVS), 2025), ClinVar (ClinVar: National Center for Biotechnology Information, 2025), OMIM (OMIM: Online Mendelian Inheritance in Man, 2025) and HPO (Human Phenotype Ontology Consortium, 2025) identifiers, as well as semantic relations, makes it well suited to extracting genetic disease information (Li et al., 2020).

A particularly important comparison is that with the NEREL-BIO (Loukachevitch et al., 2023) corpus, one of the most comprehensive resources for named entity recognition (NER) and relation extraction (RE) in biomedical texts. While the GENEXOM corpus uses a multi-level annotation structure similar to that of NEREL-BIO (Loukachevitch et al., 2023), it differs significantly in terms of its subject area and data type. NEREL-BIO (Loukachevitch et al., 2023) is based primarily on scientific publications, whereas GENEXOM is developed using real and synthetically expanded clinical reports, which gives it high practical relevance.

To improve coverage of rare pathologies and hereditary forms, the corpus was expanded to include 5,000 synthetic conclusions created using data from international databases such as OMIM (OMIM: Online Mendelian Inheritance in Man, 2025) and ClinVar (ClinVar: National Center for Biotechnology Information, 2025), and validated by geneticists. This eliminated the imbalance between categories and subtypes, thereby increasing the completeness of the annotation of rare phenotypes and nosological forms.

GENEXOM continues the tradition of the BioCreative V CDR (Li et al., 2016) and CRAFT (Bada et al., 2012) corpora, but is the first to apply their methodology to a Russian-language clinical context. Particular attention was paid to formalizing clinical dependencies such as VARIANT → GENE → DISEASE → SIGNIFICANCE, thereby ensuring compatibility with international standards [HGVS (Human Genome Variation Society (HGVS), 2025), ClinVar (ClinVar: National Center for Biotechnology Information, 2025), OMIM (OMIM: Online Mendelian Inheritance in Man, 2025) and HPO (Human Phenotype Ontology Consortium, 2025)].

Furthermore, GENEXOM builds upon the traditional Named Entity Recognition (NER) annotation framework by incorporating entities that are specific to genetic diagnostics, such as ZYGOSITY, SIGNIFICANCE, OMIM_ID, CLINVAR_ID, RECOMMENDATION and THERAPY_RECOMMENDATION, as well as semantic relations that reflect pathogenetic and diagnostic logic. This expansion makes the GENEXOM corpus unique among existing Russian-language biomedical resources.

The developed annotation framework forms the basis for subsequent stages, including relationship extraction (RE), pathogenicity classification and automated conclusion interpretation, opening up the prospect of building intelligent systems for medical genetics.

The discussion of methodological evolution has been simplified to focus on experimentally validated results presented in this study.

Future research will explore the application of instruction-tuned models and extended architectures for improving long-context reasoning and rare relation detection.

## 6 Conclusion

The scientific contribution of this study is twofold. Firstly, it developed the first multi-level annotation framework for extracting genetic disease information from Russian-language clinical reports. Secondly, it created a corresponding annotated corpus, GENEXOM.

The proposed framework encompasses formalized semantic relationships between key entities, including GENE, VARIANT, DISEASE, SIGNIFICANCE, ZYGOSITY, PHENOTYPE, INHERITANCE_MODE, OMIM_ID, CLINVAR_ID, and RECOMMENDATION. This facilitates a structured and reproducible representation of exome sequencing data.

The GENEXOM annotation scheme has proven to be highly adaptable, having been successfully applied to various languages and domains. This includes English-language genomic databases, such as ClinVar and DECIPHER, as well as national registries.

From an applied perspective, the study’s results can be used to automate genetic data analysis, support clinical decision-making, and develop intelligent systems for interpreting sequencing results.

Subsequent endeavours will concentrate on the training and evaluation of models for automatic entity and relationship extraction, the development of semi-automated annotation tools, and the integration of the proposed framework into clinical analytics platforms.

A significant finding of the study is the validation of the efficacy of the multi-level approach in comparison to conventional annotation methods, which are constrained to a rudimentary extraction of entities.

The GENEXOM project introduces a comprehensive, reproducible framework for biomedical information extraction from Russian-language clinical genetics texts. Through multi-level annotation and integration of domain ontologies [HGVS (Human Genome Variation Society (HGVS), 2025), ClinVar (ClinVar: National Center for Biotechnology Information, 2025), OMIM (OMIM: Online Mendelian Inheritance in Man, 2025), HPO (Human Phenotype Ontology Consortium, 2025)], it enables accurate recognition of entities and relations relevant to genetic diagnostics.

The proposed framework establishes a reproducible methodology for biomedical information extraction from Russian clinical genetics texts. By integrating multi-level annotation with established ontologies (HGVS, OMIM, ClinVar, HPO), the corpus enables accurate recognition of clinically meaningful entities and relations.

## Data Availability

The original contributions presented in the study are included in the article/supplementary material, further inquiries can be directed to the corresponding authors.
